# Arritmias y Riesgo de Muerte Súbita en Cardiomiopatía Hipertrófica

**DOI:** 10.47487/apcyccv.v1i2.41

**Published:** 2020-06-29

**Authors:** Jorge Salinas-Arce, Ana Cecilia Gonzales-Luna, Mario Cabrera-Saldaña, Pablo Mendoza-Novoa, Raúl Alca-Clares, Paula Solorzano-Altamirano, Freddy Del Carpio-Muñoz

**Affiliations:** 1 Unidad de Arritmias. Clínica Delgado. Lima, Perú. Unidad de Arritmias Clínica Delgado Lima Perú; 2 Unidad de Arritmias. Clínica San Felipe. Lima, Perú. Unidad de Arritmias Clínica San Felipe Lima Perú; 3 Unidad de Arritmias. Hospital Edgardo Rebagliati. Lima, Perú. Unidad de Arritmias Hospital Edgardo Rebagliati Lima Perú; 4 Unidad de Arritmias. Servicio de Cardiología Invasiva. Instituto Nacional Cardiovascular - INCOR EsSalud. Lima, Perú. Unidad de Arritmias Servicio de Cardiología Invasiva Instituto Nacional Cardiovascular - INCOR EsSalud Lima Perú; 5 Médico residente de Cardiología. Hospital Nacional Cayetano Heredia. Lima, Perú. Hospital Nacional Cayetano Heredia Lima Perú; 6 Profesor asistente. Mayo Clinic School of Medicine, Rochester, Minessota, EEUU. Mayo Clinic College of Medicine Mayo Clinic School of Medicine, Rochester Minessota USA

**Keywords:** muerte súbita, arritmias, cardiomiopatía hipertrófica, sudden death, arrhythmias, hypertrophic cardiomyopathy

## Abstract

El riesgo de muerte súbita en la cardiomiopatía hipertrófica está relacionado a la presencia de arritmias ventriculares en la gran mayoría de los casos. Es y será un reto para la Cardiología moderna encontrar los mejores esquemas para valorar la probabilidad de complicaciones arrítmicas, siendo por ahora el enfoque multifactorial la mejor estrategia para evitar el implante innecesario de dispositivos como los cardiodesfibriladores.

Si bien, el electrocardiograma (ECG) sigue siendo una excelente herramienta para el diagnóstico, incluso en fases sin expresión ecocardiográfica, no tiene un rol claro en la estratificación de riesgo. Sin embargo, la asociación de cambios en el ECG y otros hallazgos como la presencia de preexcitación y/o QT largo, así como variantes de presentación tipo cardiomiopatía hipertrófica apical, permitirían identificar a pacientes con alta probabilidad de muerte súbita.

Desde hace unos años la resonancia cardíaca y la cuantificación de la fibrosis intramiocárdica (mecanismo básico de las arritmias ventriculares) tienen un rol cada vez más importante en la evaluación de estos pacientes.

En especial, la población pediátrica debe tener una visión individualizada por el mal pronóstico a edades tempranas y por el rol incierto de diferentes herramientas para la valoración del riesgo y tratamiento.

## Abstract (en)

The risk of sudden death in hypertrophic cardiomyopathy is related to the presence of ventricular arrhythmias in most cases. Finding the best schemes to assess the probability of arrhythmic complications will remain a challenge for modern Cardiology. Meanwhile, the multifactorial approach is the best strategy to avoid the unnecessary implantation of devices such as the implantable cardioverter defibrillator.

Although the electrocardiogram remains an excellent diagnostic tool, even before echocardiographic expression, it does not have a clear role as a risk factor. However, the identification of associated arrhythmias such as preexcitation or long QT and variants of presentation as apical hypertrophic cardiomyopathy, allows identifying patients with high probability of sudden death.

During the last few years, cardiac resonance and quantification of intramyocardial fibrosis (the basic mechanism of ventricular arrhythmias) have gained an important role in the evaluation of these patients.

In particular, pediatric patients must have an individualized approach due to the poor prognosis at early ages and the uncertain role of different tools for risk assessment and treatment.

## Introducción

Han pasado más de 60 años desde la descripción de 8 casos de “tumor cardíaco primario” asociado a muerte súbita (MS), que en la anatomía patológica correspondieron a hipertrofia ventricular asimétrica o “hamartomas musculares” como se les denominó en ese momento.^1^

La cardiomiopatía hipertrófica (CMH) es una cardiopatía genética debida a mutaciones en genes codificadores de las proteínas del sarcómero. Se estima que su prevalencia en la población general es al menos de 1:500 individuos, convirtiéndola en la cardiopatía monogenética más frecuente.^2^ En esta revisión discutiremos la relación entre las arritmias cardíacas y la MS en el paciente con CMH, con especial énfasis en la estratificación de riesgo, el rol del electrocardiograma y las particularidades del paciente pediátrico. 

## Epidemiología de las Arritmias y la Mortalidad en la CMH

Las arritmias supraventriculares y ventriculares están presentes casi en la totalidad de pacientes con CMH. Diferentes estudios basados en monitorización *Holter* de 24-48h describen extrasístoles ventriculares en 80-95% de los pacientes e incluso episodios de taquicardia ventricular (TV) no sostenida son encontrados en el 20-30%.^3^^)^

Si bien la presencia de arritmias ventriculares establece un potencial riesgo de complicaciones, este riesgo no es absoluto y depende de la asociación con otros factores. De hecho, es importante recordar que las causas de mortalidad no se restringen a eventos cardíacos súbitos por arritmias, sino además, a insuficiencia cardíaca (IC) terminal y eventos tromboembólicos asociados a fibrilación auricular (FA).^4^

Inicialmente, el riesgo de muerte en el paciente con CMH fue descrito hasta de 6% anual, posteriores análisis de la historia natural de la enfermedad, calcularon dicho riesgo alrededor de 1-2% anual.^5^ Con la institución del cardiodesfibrilador implantable (CDI), trasplante cardíaco y tratamiento de FA, la mortalidad asociada a CMH está alrededor de 0.5%, muy similar a la mortalidad de la población general sin cardiomiopatía.

## Fibrosis Miocárdica y Fisiopatología de las Arritmias Ventriculares

La fibrosis miocárdica (FM) es un hallazgo distintivo de la CMH y es uno de los mayores contribuyentes al riesgo de arritmias ventriculares, disfunción ventricular, IC y MS. En pacientes con enfermedad manifiesta, el examen histopatológico revela la presencia de diferentes grados de FM, y hasta en dos tercios de pacientes esta puede ser detectada y cuantificada mediante la resonancia magnética con contraste.^6^

**Figura 1:**
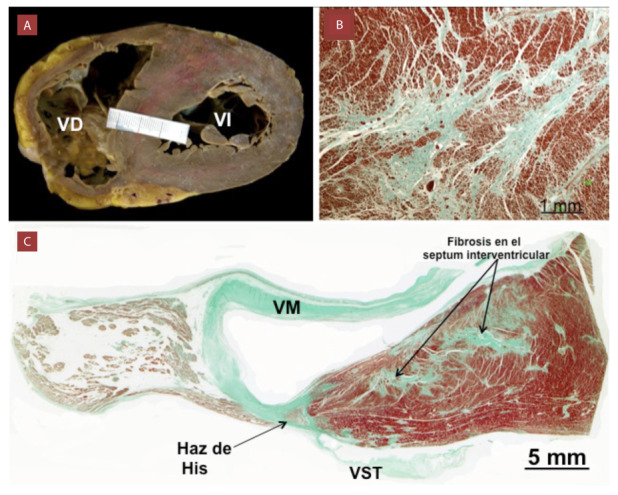
Estudio anatomo-patológica de una mujer de 42 años con miocardiopatía hipertrófica.

En la CMH se observan dos tipos de FM: la fibrosis intersticial que se desarrolla alrededor de los cardiomiocitos y que se puede encontrar en los estadios incipientes de la enfermedad, incluso antes de la manifestación fenotípica definida (hipertrofia ventricular); y fibrosis densa que reemplaza a los cardiomiocitos dañados o muertos, similar a la escara ventricular que se observa en la cardiomiopatía isquémica post infarto de miocardio. Esta fibrosis densa se relaciona al proceso de reparación de los cardiomiocitos, en un inicio como expresión de las mutaciones sarcoméricas y más tarde por los cambios patológicos que incluyen la obstrucción intracavitaria, la enfermedad isquémica de pequeños vasos y la isquemia miocárdica.^7^ (Figura 1) 

A) Corte transversal que muestra hipertrofia septal desproporcionada en relación a la pared libre del ventrículo izquierdo (VI). B) Se observa histológicamente desorganización de las células musculares cardíacas y aumento de la matriz del tejido conectivo (colágeno en color verde) produciendo cicatrices miocárdicas. Además, arterias coronarias intramiocárdicas de pequeño calibre. (Tinción: tricrómico de Masson) C) Sección histológica a nivel del haz penetrante de His. Nótese como el haz de His está rodeado por completo por tejido conectivo y se observan acúmulos fibróticos a nivel del septum interventricular. (Tinción: tricrómico de Masson) Colaboración: Damián Sánchez-Quintana Md. PhD. Departamento de Anatomía y Biología Celular, Facultad de Medicina, Universidad de Extremadura, Badajoz, España. En: El electrocardiograma y el diagnóstico Clínico. Segunda Edición. Editor Jorge Salinas-Arce. 2014 VD: ventrículo derecho; VM: válvula mitral; VST: velo septal de la válvula tricúspide.

El mecanismo subyacente de las taquiarritmias ventriculares está asociado a los cambios estructurales característicos de la enfermedad. Primero, la desorganización histológica de los cardiomiocitos puede producir variaciones en el tiempo de conducción y periodo refractario, lo cual incrementa el riesgo de formación de circuitos de reentrada y el riesgo de desarrollar TV o fibrilación ventricular (FV) sostenida. Segundo, la presencia de isquemia miocárdica por el desbalance entre oferta y demanda de oxígeno en áreas de hipertrofia marcada o isquemia debido a la presencia de enfermedad coronaria microvascular. Tercero, la presencia de FM y su conocido rol en la arritmogénesis ventricular, de manera parecida a la observada en las cardiomiopatías isquémica y dilatada. Finalmente, algunos pacientes progresan a una fase avanzada de deterioro y remodelación de la función ventricular *(burnout phase)* caracterizada por dilatación y caída marcada de la función ventricular.8^7^^,^^8^

Las arritmias causantes de MS en pacientes con CMH son la FV y la TV sostenidas, esta información ha sido confirmada mediante el análisis de la interrogación de CDIs en pacientes con CMH y eventos arrítmicos. Basado en los registros de los CDI, la TV monomórfica rápida fue la taquiarritmia ventricular más frecuente y en su mayoría fue desencadenada por extrasístoles ventriculares con intervalos de acoplamiento largo.^8^

## La Estratificación del Riesgo de Muerte Súbita y sus Limitaciones

Por distintas razones, la estimación actual del riesgo de MS en la CMH tiene varias limitaciones. Primero, todos los estudios en los cuales se basan las recomendaciones son descriptivos (muchos retrospectivos) y todos los factores de riesgo identificados no han sido validados en estudios aleatorizados. Segundo, debido al bajo riesgo promedio de MS y la baja frecuencia de eventos, los análisis estadísticos multivariable y la construcción de modelos de predicción son muy limitados; esto hace que el valor predictivo positivo de cualquier factor de riesgo sea bajo. Tercero, la definición de los factores de riesgo varía de manera amplia en diferentes estudios, incluso los que son aceptados en consenso. Cuarto, hay una tendencia a disociar ciertos factores de riesgo (presente versus ausente), cuando la introducción de gradualidad o se-veridad sería más informativo y más predictivo. ^8^^)^ A continuación, desarrollamos los principales factores de riesgo y la evidencia científica en la cual se basa la estratificación del riesgo.

### 1. Historia personal de MS cardíaca, fibrilación o taquicardia ventricular sostenida

Es el factor de riesgo más importante, y está considerado como una clara indicación para implantar un CDI como medida de prevención secundaria. En este grupo de pacientes el riesgo de recurrencia de MS es 40-45% en cinco años o cerca al 10% anual. ^4^ La historia personal incluye episodios documentados de taquiarritmias ventriculares sostenidas. En el seguimiento a largo plazo, los pacientes con CDI por prevención secundaria, presentan eventos arrítmicos y descargas apropiadas recurrentes que tienden a agruparse temporalmente y pueden presentarse después de periodos silentes prolongados de hasta décadas. Se desconoce la razón por la cual el substrato arritmogénico en la CMH puede manifestarse en determinados periodos de la enfermedad y puede permanecer latente o inactivo por muchos años, y se postula que una inestabilidad autonómica podría estar involucrada. ^8^

### 2. Historia familiar de MS cardíaca y de cardiomiopatía hipertrófica

El hallazgo de grupos familiares con CMH con una frecuencia alta de MS planteó la posibilidad de la existencia de un factor familiar que predispone a estos eventos. Sin embargo, en la actualidad no hay consenso para considerar a la historia familiar de MS como factor individual de eventos fatales. Además, las definiciones equivalentes a “historia familiar positiva” también han sido variables en diferentes estudios. De acuerdo a las guías americanas, se considera la historia familiar de MS debido a CMH en al menos un familiar de primer grado sin especificar la edad.^9^ En tanto, las guías europeas definen la historia familiar como MS cardíaca en uno o más familiares de primer grado menores de 40 años sin requerir de un diagnóstico definitivo de CMH, o MS cardíaca en un familiar de primer grado a cualquier edad con un diagnóstico establecido de CMH. ^10^ Bos *et al*. ^11^^)^ encontraron en pacientes con historia familiar de MS como único factor de riesgo, una mortalidad cercana a 10%, con una frecuencia anual de eventos de 2.2%. 

### 3. Síncope no explicable 

Los episodios de síncope en pacientes con CMH son frecuentes. En estudios poblacionales relacionados a factores de riesgo de MS, uno de cada cinco pacientes tuvo algún episodio de síncope, mientras que en centros especializados de referencia hasta uno de cada tres pacientes tuvo una historia de síncope. El síncope relacionado a mayor riesgo de muerte súbita es el síncope que no tiene explicación clínica y tiene características de etiología arrítmica ventricular. ^12^ En una serie de 1,511 pacientes con CMH, 14% tuvieron episodios de síncope, incluyendo síncope no explicable en el 10%; después de un seguimiento de casi 6 años, el riesgo relativo de MS fue 1.8 veces mayor en los pacientes con síncope no explicable comparado con los pacientes que no presentaron síncope, y los pacientes con síncope vasovagal tuvieron el mismo riesgo de MS que los pacientes que no tuvieron síncope. ^13^ La presencia de síncope no explicable en pacientes jóvenes indica un riesgo mucho mayor; en los pacientes con síncope de etiología no precisa menores de 18 años, la mortalidad por MS fue más alta, hasta un 60% a los 5 años y un riesgo 8 veces mayor. ^13^

### 4. Hipertrofia ventricular izquierda masiva (grosor máximo > 3 cm)

La hipertrofia ventricular izquierda es la manifestación clínica más esperada de la CMH. Basado en este concepto, se podría inferir que un paciente con hipertrofia ventricular masiva (HVM), tiene una expresión fenotípica mayor y entonces debe estar asociado a un peor pronóstico, incluyendo un mayor riesgo de desarrollar arritmias ventriculares y MS. Elliott *et al.*^14^ en una población de 630 pacientes con CMH evaluados por ecocardiografía, prueba de esfuerzo y monitoreo electrocardiográfico ambulatorio, tras un seguimiento medio de 59 meses, describieron que 39 pacientes murieron súbitamente o tuvieron una terapia del CDI apropiada; aunque hubo tendencia hacia mayor riesgo de eventos en pacientes con mayor grosor de la pared del VI, la mayoría de eventos ocurrió en pacientes con un grosor de la pared menor a 30 mm (sólo 10 de los 39 pacientes con MS tuvieron HVM) y, la acumulación de factores de riesgo adicionales tuvo mucho mayor valor predictivo que la sola presencia de hipertrofia masiva del VI; así, se entiende que la simple ausencia de HVM fue insuficiente para garantizar un buen pronóstico del paciente individual. 

En un estudio más reciente realizado por Olivotto *et al.,*^15^ luego de un seguimiento medio de 12 años, 16 pacientes presentaron MS y el grosor máximo del VI no estuvo asociado con el riesgo de MS. Además estos investigadores encontraron que en pacientes jóvenes, la presencia de HVM tiene mayor implicancia pronóstica, pero no debe ser tomado en cuenta en forma aislada, sino más bien, en el contexto de un análisis multifactorial. Este concepto es resaltado en las recomendaciones de las Guías Europeas para CMH, en las cuales la presencia de hipertrofia VI > 3 cm no es un criterio tipo I para el implante de un CDI. ^10^

### 5. Taquicardia ventricular no sostenida

En comparación a la TV sostenida, los episodios de TV no sostenida (TVNS) acarrean un valor predictivo menor de MS en pacientes con CMH. Se define como TVNS la presencia de al menos 3 complejos ventriculares consecutivos, a una frecuencia mayor de 120 por minuto, duración no mayor a 30 segundos, sin deterioro hemodinámico y documentado mediante monitoreo ambulatorio por un periodo de 24-48 horas. ^10^

La presencia de TVNS asintomática, documentada en monitoreo ambulatorio electrocardiográfico es bastante frecuente y ocurre en hasta un cuarto de pacientes con CMH, e incluso es más frecuente si el tiempo de monitoreo se incrementa. Es típico durante periodos de tono vagal incrementado y está asociado a un riesgo mayor de MS, en particular en pacientes jóvenes.

Uno de los estudios más relevantes que evaluó este factor de riesgo incluyó 532 pacientes con CMH, todos con *Holter* por 24 a 48 horas, de los cuales casi el 20% tuvieron episodios de TVNS. Se observó una interacción entre la presencia de TVNS y la edad. Después de 5 años, el 25% de pacientes menores de 20 años tuvieron eventos de MS y la presencia de TVNS incrementó 4.4 veces el riesgo de MS en pacientes jóvenes, mientras que, el riesgo en pacientes mayores fue 2.3 veces mayor comparado con pacientes que no tuvieron TVNS, aunque en este último grupo no se consiguió significancia estadística. Cabe resaltar que la duración, frecuencia de episodios o la frecuencia cardíaca durante los episodios de TVNS, no acarreó ningún factor pronóstico. ^16^

### 6. Respuesta anormal o atenuada de la presión arterial durante la prueba de esfuerzo

Se considera una respuesta anormal, cuando la presión arterial (PA) sistólica incrementa menos de 20 mmHg en la fase más intensa del ejercicio comparada con la presión sistólica de base o cuando hay una caída de al menos 20 mmHg durante el ejercicio. ^10^ Aproximadamente un tercio de pacientes con CMH tiene una respuesta anormal o atenuada de la PA al ejercicio, caracterizada por hipotensión progresiva o inhabilidad de incrementar la PA. Este factor de riesgo ha sido asociado a mayor riesgo de MS sobretodo en pacientes jóvenes menores de 40 años y no tiene valor pronóstico en pacientes mayores. ^8^^,^^10^

## La Fibrosis Miocárdica y la Resonancia Magnética Cardíaca

El rol de la resonancia magnética (RM) en la evaluación de las cardiomiopatías es cada vez más importante y puede ser usada para definir el diagnóstico de CMH en pacientes con ecocardiogramas ambiguos o en quienes no se puede evaluar áreas de hipertrofia debido a ventanas acústicas inadecuadas. La RM tiene un rol especial en la diferenciación de CMH de la hipertrofia cardíaca en atletas o enfermedad hipertensiva. Así mismo, la RM es el método de elección para evaluar la extensión de la fibrosis miocárdica al usar sustancias de contraste quelante (gadolinio). 

Varios estudios han evaluado la extensión de la fibrosis miocárdica como predictor del riesgo de MS en CMH y dos de ellos son los más relevantes. El primero por Cha *et al*. ^17^ reportó que la fibrosis miocárdica extensa es un predictor robusto de MS; este estudio incluyó 1,293 pacientes con CMH de los cuales 23 murieron de súbito o sobrevivieron un paro cardíaco y 17 tuvieron descargas apropiadas del CDI durante un seguimiento prospectivo de 3.3 años después del estudio de RM. Los autores describen una relación continua y directa entre la extensión de fibrosis y el riesgo de los eventos de MS o terapias del CDI. Así los pacientes con fibrosis de al menos 15% de la masa total de miocardio tuvieron dos veces más riesgo de MS o descargas del CDI con una frecuencia de eventos de 6% a los 5 años. Mientras tanto, la ausencia de fibrosis se asoció con casi 60% de reducción del riesgo. El segundo estudio por Ismail *et al*. ^18^ incluyó 711 pacientes con CMH que tuvieron una RM de base; aproximadamente dos tercios de pacientes tuvieron fibrosis miocárdica identificable por RM y 21 pacientes (3.1%) tuvieron MS o MS abortada después de un seguimiento de 3.5 años. En contraste con el estudio anterior, la extensión de fibrosis miocárdica no estuvo asociada con los eventos de interés después de ajustar el análisis multivariable e incluir la fracción de eyección del VI, mientras que esta última sí estuvo relacionada con los eventos de MS. Para conciliar los resultados contradictorios de estos estudios, un meta análisis que incluyó cinco estudios reportó una relación directa entre la extensión de fibrosis y el riesgo de MS o MS abortada. ^19^

## El Electrocardiograma y el Riesgo de Muerte Súbita

En la CMH se observan numerosas anormalidades electrocardiográficas, las mismas que pueden ser la única manifestación de la enfermedad en una etapa temprana; estas alteraciones están determinadas por la extensión, el grado, la distribución de la hipertrofia del miocardio, así como, por la presencia de fibrosis, necrosis del músculo cardíaco y, por la aparición de trastornos de la conducción intraventricular. ^9^^,^^20^

Alrededor del 95% de los pacientes con diagnóstico de CMH presentan alteraciones en el electrocardiograma (ECG), no existiendo signos patognomónicos. El patrón ECG normal, presente en 5 a 10% de casos, está asociado con formas fenotípicas menos severas y un curso cardiovascular favorable; sin embargo, el patrón ECG anormal no predice eventos de muerte súbita futura o terapia apropiada del CDI. ^9^^,^^20^

En el ECG encontramos signos de crecimiento auricular izquierdo y derecho, y prolongación de la onda P (un predictor conocido de FA), que rara vez ocurren de forma aislada. ^20^ El bloqueo completo de rama es poco común en CMH, y se observa con más frecuencia en situaciones post intervenciones terapéuticas como la miectomía o ablación septal. El bloqueo de rama izquierda puede observarse en estadios avanzados de la enfermedad, asociados frecuentemente con escara anteroseptal.

La fragmentación del QRS, definido por la presencia de onda R adicional o una muesca en el nadir de la onda S, refleja demora de la conducción intraventricular y es un marcador de fibrosis miocárdica. ^20^

Los trastornos de la repolarización son los hallazgos más frecuentes y están presentes en alrededor del 50% de los pacientes con hipertrofia ventricular izquierda (HVI). Se observan cambios en el punto J, descenso del segmento ST y onda T negativas en derivaciones izquierdas. Esta condición se denomina *strain* electrocardiográfico del ventrículo izquierdo. La presencia de *strain* electrocardiográfico se correlaciona con el incremento de la masa ventricular observada en la ecocardiografía. 

**Figura 2:**
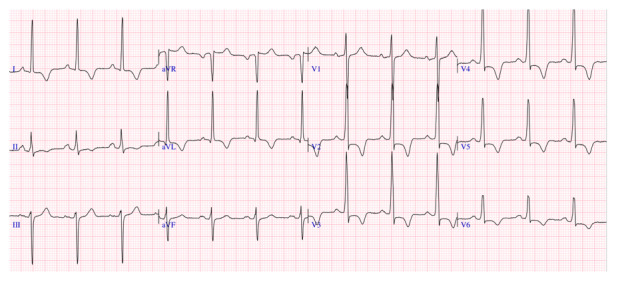
Electrocardiograma en paciente varón de 44 años con diagnóstico de CMH a predominio septal (septum 18 mm y pared posterior 11 mm) presenta: ondas R altas en precordiales V2-V4, descenso del segmento ST en precordiales (strain electrocardiográfico), QTc: 460 ms. Onda T negativa en precordiales y positiva en aVR.

La alteraciones en la onda T están presentes en la mayoría de los pacientes con CMH sintomática. Uno de los aspectos más prometedores en el rol del ECG es la observación de una onda T positiva en aVR; este hallazgo está asociado a eventos arrítmicos mayores (muerte súbita cardíaca, taquicardia ventricular sostenida, fibrilación ventricular o terapias apropiadas por el CDI), independiente de los factores de riesgo tradicionales. ^21^^)^ (Figura 2)

La prolongación del intervalo QT está presente hasta en el 63.2% de casos, existiendo correlación entre el grosor máximo de la pared del VI y el intervalo QTc prolongado. Recientemente, se describió que la prolongación de QTc es un predictor clínico de terapias apropiadas por el CDI en pacientes con CMH. ^22^

El ECG es extremadamente útil para el diagnóstico diferencial de CMH sarcomérica y sus fenocopias. Mientras que las ondas Q con ondas T positivas en las mismas derivadas (discordancia Q / T) y ondas T profundas gigantes (>10 mm) en las derivadas anterolaterales apoyan el diagnóstico de CMH sarcomérico; el intervalo PR corto y(o) la prolongación de la deflección intrinsecoide son hallazgos comunes en enfermedades de depósito y enfermedad mitocondrial. ^20^

A largo plazo, las enfermedades por depósito y las mitocondriales pueden progresar a retraso de la conducción auriculoventricular (AV) y, en última instancia, a bloqueo AV de alto grado; esta última también es una complicación común de los trastornos infiltrativos como la amiloidosis cardíaca. Las enfermedades de depósito están invariablemente acompañadas por complejos QRS de alta amplitud con criterios de voltaje para HVI con depresión del segmento ST y ondas T profundas e invertidas en derivadas laterales. Y, por el contrario, la expansión del intersticio miocárdico secundario a la infiltración típicamente causa bajos voltajes QRS en la amiloidosis.^20^

Incluso utilizando estratificaciones de riesgo con múltiples criterios electrocardiográficos, en la actualidad, el ECG no es utilizado en la evaluación del pronóstico y tratamiento de los pacientes con CMH. 

## Cardiomiopatía Hipertrófica Apical

La CMH apical es una forma poco frecuente de CMH, observada hasta en 5% de los casos que, en muchas oportunidades es considerada como una forma “benigna”; sin embargo, tiene un riesgo alto de complicaciones arrítmicas y MS cardíaca, en especial, si se asocia a aneurisma apical y a un incremento del gradiente medio ventricular.^23^

**Figura 3:**
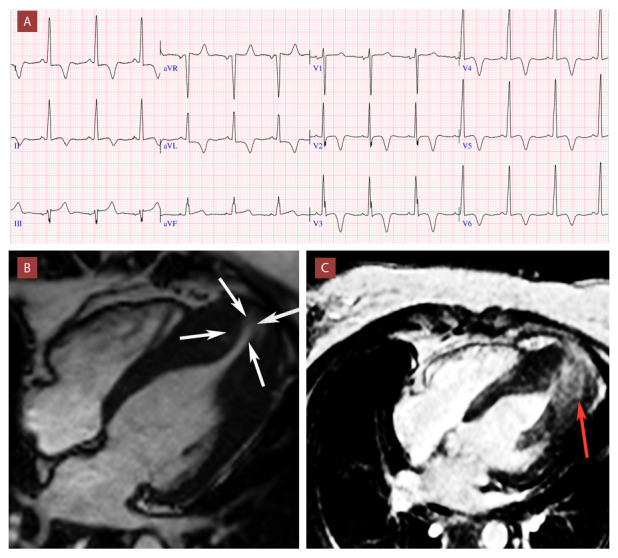
Mujer de 41 años con diagnóstico de cardiomiopatía hipertrófica apical (espesor máximo 23 mm) asociada a aneurisma y patrón obstructivo medio ventricular.

El ECG típico de esta CMH fue descrito por Yama-gushi,^24^ presentando ausencia de la onda Q en I, aVL y en V5-V6, presencia de ondas R altas desde las derivadas V2, segmento ST rectificado y ascendente fundamentalmente en la derivada V2, y ondas T negativas “gigantes” “pseudo-isquémicas” en la cara anterolateral (> 10 mm).^24^ (Figura 3) Otros hallazgos como la presencia de elevación del segmento ST y fragmentación del QRS en derivadas de la pared inferior han sido asociados a la presencia de aneurisma apical y fibrosis miocárdica.

A) Electrocardiograma. Onda S profunda en V1 y onda R alta en V5; ondas T negativas (- 8 mm en V3) desde V2 a V6, DI y aVL; ausencia de ondas Q en derivadas laterales; elevación del punto J (1 mm) en DIII y aVF, con patrón rR´ en DIII. B) Resonancia magnética cardíaca. Vista de cuatro cámaras. Aneurisma apical de 17 mm, sin presencia de trombo. La hipertrofia apical se extiende hasta la base de los músculos papilares con obstrucción medioventricular por coaptación de la pared anterolateral y septal en sístole (flechas blancas) C) Realce tardío de gadolinio (RTG). Se observa un patrón de RTG no isquémico parcheado localizado en ápice (flecha roja) y borde aneurismático con extensión a áreas contiguas de la porción distal de pared anterolateral e inferoseptal.

El desarrollo de aneurisma apical en algunos pacientes con CMH (apical y no apical, como en CMH obstructiva medio ventricular) es descrito en el 2.2 a 8.5 % de los casos, en su mayoría asociados a importantes complicaciones cardiovasculares. Hallazgos en el ECG como la elevación del segmento ST ≥ 1 mm de V3 a V5, con una sensibilidad del 66.7% y especificidad del 98.7%, permiten identificar los casos en una fase más temprana. ^25^

Es importante considerar esta forma de presentación de la cardiomiopatía hipertrófica debido a que muchas veces es considerada como de bajo riesgo y, de hecho, los esquemas de estratificación actuales no la consideran dentro de sus criterios.

## Cardiomiopatía Hipertrófica asociada a Otras Arritmias

La FA es la arritmia más frecuente en la CMH, con una prevalencia hasta de un 28%. La presencia de esta arritmia está asociada a un riesgo mayor de eventos isquémicos, IC y MS; así, Siontis *et al.*^26^ encontraron un incremento en la mortalidad total de los pacientes con CMH asociada a FA (HR: 1.48; IC95%: 1.27-1.71). 

La asociación de arritmias diferentes a la FA y CMH fue descrita por primera vez en el año 1985 en pacientes con síndrome de Wolff Parkinson White (WPW), alteraciones en la conducción AV y CMH.^27^ Con los años esta asociación se denominaría síndrome de PRKAG2, siendo en realidad una cardiomiopatía de depósito de glucógeno, caracterizada por la triada de: síndrome de WPW, hipertrofia ventricular y trastorno del sistema de conducción, siendo uno de los diagnósticos diferenciales a tener en cuenta, en especial si las manifestaciones clínicas están presentes desde edades muy tempranas. ^28^

**Figura 4:**
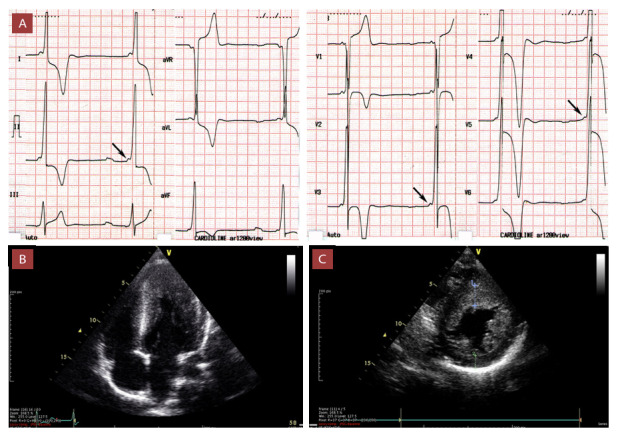
Varón de 15 años, futbolista pre profesional, en evaluación por dolor torácico, palpitaciones y pre síncope.

En pacientes con CMH y WPW, sin una causa genética estudiada o establecida, existe mayor riesgo de episodios sincopales y eventos de FA. Se conoce que la presencia de vías accesoria está asociado a episodios de FA paroxística en ausencia de otra patología.^29^ (Figura 4)

A) Electrocardiograma en ritmo sinusal con PR 90 ms presencia de onda delta positiva en derivadas inferiores y precordiales, complejos QRS de gran amplitud, descenso del ST V3-V6 y onda T negativas profundas de V2-V6. B) Ecocardiografía transtorácica vista apical 4 cámaras muestra hipertrofia ventricular. C) Ecocardiografía transtorácica vista paraesternal eje corto permite observar hipertrofia ventricular asimétrica a predominio septal con máximo espesor 21 mm. La pared posterior mide 14 mm. En el estudio electrofisiológico presentó vía accesoria fascículo ventricular.

Los pacientes con CMH pueden presentar un incremento del intervalo QT en el electrocardiograma, que además supone mayor riesgo de arritmias ventriculares malignas y muerte súbita en paciente con Síndrome de QT adquirido o congénito.^30^^,^^31^ Estudios muestran que pacientes con CMH tienen hasta un 13% de incremento del QTc por encima de 480ms, inclusive en ausencia de fármacos que prolonguen el QT.^32^ Este incremento del intervalo QTc se asocia probablemente a estadios más severos de CMH, con mayor gradiente obstructivo, peor clase funcional y mayor grado de hiper-trofia. No existen datos con respecto a qué valor de QTc esté relacionado con riesgo de MS, por lo que las recomendaciones se basan en realizar medidas continuas del intervalo QT en cada atención de los pacientes y seguir utilizando el valor de QTc mayor de 500 ms como riesgo arrítmico pero contrastando con el escenario clínico de cada paciente.^31^

## Cardiomiopatía Hipertrófica y Arritmias en la Edad Pediátrica

Las miocardiopatías hipertróficas en edad pediátrica son entidades de infrecuente presentación, con una incidencia de 0.47 por 100,000 pacientes por año en el registro más grande de EEUU.^33^ Si bien las características morfológicas y clínicas son similares a las del adulto, el pronóstico difiere. La mortalidad en lactantes a los 2 años del diagnóstico es de 30%, mientras que los diagnosticados en edad escolar o mayores tienen mayor sobrevida. La muerte súbita también es rara en esta entidad cuando no se asocia a otras alteraciones genéticas 

**Figura 5:**
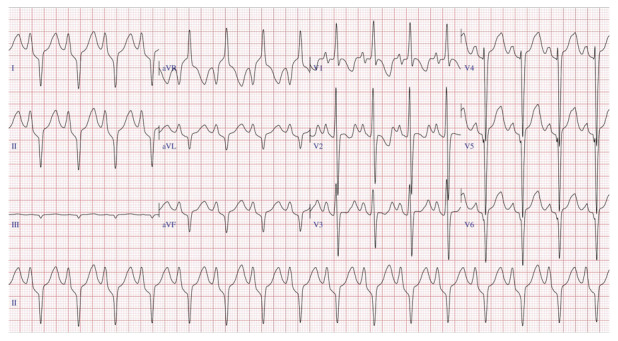
Electrocardiograma en ritmo sinusal de paciente de 6 meses de edad con síndrome de Noonan y miocardiopatía hipertrófica biventricular obstructiva.

El diagnóstico fenotípico se realiza a través de ecocardiograma, y el EKG tendría un valor predictivo negativo mayor del 99%.^34^ El crecimiento auricular producto de la disfunción diastólica suele provocar arritmias atriales: extrasístoles frecuentes, taquicardia atrial y en casos más avanzados FA. (Figura 5)

Alteración de la relación R/S en precordiales y ondas Q en derivadas inferiores y V5-V6. Los cambios en el eje son atípicos pero explicados por el predominio de las fuerzas derechas debido a la mayor masa ventricular en el septum medio y en la porción media de la pared libre del VD.

A diferencia del adulto, en los niños los fenotipos no dependientes del sarcómero tienen muchas más etiologías, incluyendo las conocidas RASopatías (siendo el principal exponente el síndrome de Noonan), los desórdenes metabólicos de depósito (como la enfermedad de Pompe), los desórdenes neurodegenerativos (ataxia de Friedreich) y desórdenes mitocondriales.^35^ Las presentaciones sindrómicas suelen asociarse a diagnósticos en edades más tempranas y se presentan como alteraciones neurológicas y musculoesqueléticas. 

La sobrevida es buena cuando el diagnóstico se hace después del primer año de vida, llegando al 97 % a los 5 años y al 94% a los 10 años del diagnóstico. El primer pico de mortalidad es antes del año de vida y el segundo entre los 8 y 12 años, y la causa principal de muerte es la falla cardíaca. Es importante resaltar que el 36% de MS pediátricas tienen como etiología la CMH, contra el 3% por miocardiopatías dilatadas. ^36^

Los métodos para estratificar población adulta no han sido validados en niños. Ni la RM con evaluación del reforzamiento tardío, ni la elevación de biomarcadores se han podido usar como métodos fiables para la estratificación de riesgo de MS. 

Los betabloqueadores son el tratamiento de elección en sintomáticos. Se ha descrito una necesidad de dosis más altas de las habituales para el manejo de falla cardíaca por componente obstructivo, llegando hasta 24 mg/kg/día de propranolol o 5.9 mg/kg/día de metoprolol, en ambos casos sin aparente efecto negativo en calidad de vida y capacidad de ejercicio. ^37^^,^^38^ En caso de intolerancia al betabloqueador se plantea reemplazarlo por calcio antagonistas como el verapamilo, aunque este último está asociado a riesgo de MS en pacientes con hipertrofia severa. ^39^ En pacientes con síntomas refractarios, la disopiramida ha demostrado, en pequeñas series, mejoría tanto de síntomas como de gradientes a dosis iniciales de 6 mg/kg/d hasta 20 mg/kg/d, con vigilancia del intervalo QT, pudiéndose indicar incluso en combinación con betabloqueadores.^39^

Si bien la MS es rara antes de la adolescencia, cuando se considera el implante de un CDI nos enfrentamos a una decisión difícil, en especial, por la falta de evidencia para la selección de los pacientes. Ninguno de los factores de riesgo en adultos ha sido validado en niños; sin embargo, la Sociedad Europea recomienda el implante de CDI con dos factores mayores de riesgo mientras que la Sociedad Americana recomienda el implante con la presencia de un solo factor de riesgo.^35^^,^^40^^)^

## Financiación:

Ninguna
